# Techniques to Study Autophagy in Plants

**DOI:** 10.1155/2009/451357

**Published:** 2009-08-27

**Authors:** Géraldine Mitou, Hikmet Budak, Devrim Gozuacik

**Affiliations:** Biological Science and Bioengineering Program, Faculty of Engineering and Natural Sciences, Sabanci University, Orhanli, Tuzla 34956, Istanbul, Turkey

## Abstract

Autophagy (or self eating), a cellular recycling mechanism, became the center of interest and subject of intensive research in recent years. Development of new molecular techniques allowed the study of this biological phenomenon in various model organisms ranging from yeast to plants and mammals. Accumulating data provide evidence that autophagy is involved in a spectrum of biological mechanisms including plant growth, development, response to stress, and defense against pathogens. In this review, we briefly summarize general and plant-related autophagy studies, and explain techniques commonly used to study autophagy. We also try to extrapolate how autophagy techniques used in other organisms may be adapted to plant studies.

## 1. Introduction

Autophagy, literally meaning self (auto) eating (phagy), is an evolutionarily conserved and highly regulated catabolic process that leads to the degradation of cellular components using lysosomal/vacuolar degradation machinery of the same cell. Depending on the mechanism of transport to lysososome/vacuole, at least three forms of autophagy have been described: “Macroautophagy” is characterized by the engulfment of long-lived proteins and organelles in de novo formed double-/multimembrane vesicles called autophagosomes or autophagic vesicles. These vesicles subsequently deliver their cargo to the lysosome or vacuole for degradation. In another form of autophagy, called “microautophagy,” lysosome/vacuole directly engulfs cytosolic components through an invagination of its membrane [1, 2]. A third common form of autophagy is called “chaperone-mediated autophagy” (CMA). CMA is a very selective process during which proteins with a KFERQ consensus peptide sequence are recognized by a chaperone/cochaperone complex and delivered to the lytic compartment in an unfolded state [3, 4]. 

Macroautophagy is the most studied form of autophagy. Macroautophagy (“autophagy” hereafter) occurs at basal levels in growing cells, allowing them to recycle long-lived proteins and organelles [3]. The cargo is degraded into its building blocks (i.e., proteins to amino acids), helping the cell to economize its resources, eliminate old/damaged organelles, and survive nutrient and other types of stress. For example, in plants under conditions causing cellular and organismal stress such as starvation, drought, and other abiotic stress, autophagy is upregulated [5–8]. Autophagy is also involved in physiological phenomena including plant development, senescence, and immune response [9–11]. In some cases, autophagy can function as a nonapoptotic and alternative programmed cell death mechanism, and its role in plant cell death was explored [12–15]. As a consequence of its involvement in several important physiological and pathological phenomena, autophagy became one of the fastest expanding fields of molecular biology in recent years.

In this review, we will briefly summarize the mechanisms of autophagy in general and particularly plant autophagy, list commonly used techniques to detect and quantify autophagy, and finally discuss their utility in plant autophagy detection. An exhaustive summary of the autophagy mechanisms is beyond the scope of this review. The readers may find an in-depth discussion of the mechanistic aspects of autophagy in recently published reviews [5, 9, 16].

## 2. General Autophagy Mechanisms

**Figure 1:**
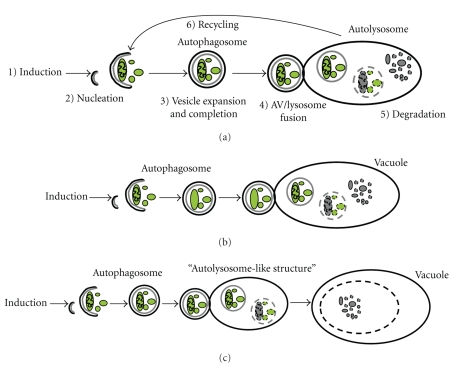
Autophagy mechanism and alternative pathways for autophagosomes in plants. (a) Following an upstream stimulus, such as starvation, double membrane vesicles, autophagosomes, appear and engulf portions of cytosol, long-lived proteins, and organelles such as mitochondria. Autophagosomes eventually fuse with lysosomes, endosomes, or vacuole. Autophagosomes are degraded together with their cargo and the building blocks are pumped back into the cytosol for reuse. (b) Autophagosomes may fuse directly with the vacuole (observed in *A. thaliana*) (c) or, may first fuse with “lysosome-like structure” or endosomes to form “autolysosome-like structures” and then, eventually may fuse with the vacuole (observed in tobacco plant).

So far, nearly 30 autophagy-related genes (depicted by the acronym *ATG*) were identified using yeast mutants [17]. Plant and mammalian orthologues of most of these genes and proteins are now characterized. Data obtained from these studies underline the fact that the basic machinery of autophagy is preserved from yeast to higher eukaryotes. Autophagy proceeds through five distinct phases: namely, induction, nucleation, vesicle expansion and completion, autophagosome/lysosome fusion, and cargo degradation [9, 18] (Figure 1(a)). 

### 2.1. Induction

This is the phase where upstream signaling mechanisms leading to autophagy activation are turned on. Many of these pathways are integrated by the “Target of rapamycin (Tor)” protein [19–21]. Tor is a serine/threonine kinase regulated in response to variation in amino acids, ATP, and growth factors. Downregulation of Tor activity correlates with autophagy stimulation [22]. Tor pathway and its effect on autophagy were preserved in plants. Yet, structural differences exist between Tor proteins in plants and other eukaryotes, therefore, rapamycin, a widely used specific inhibitor of Tor, cannot be used to study autophagy in plants [23, 24]. 

Tor inactivation induces autophagy at least by two mechanisms in yeast. The first involves activation of transcription factors called GLN13 (nitrogen regulatory protein) and GCN4 (General Control Nondepressible), leading to transcriptional upregulation of some of the *ATG* genes (e.g., *ATG1* and *ATG13*) [25, 26]. Second mechanism is related to the modification by Tor of an autophagy protein complex containing Atg1 and Atg13. Active Tor induces hyperphosphorylation of Atg13 inhibiting its association with Atg1 (AtAtg1 in *A. thaliana* and ULK1 (Unc-51-like kinase1) in mammals), a serine/threonine kinase required for autophagy [27]. Tor inactivation leads to rapid dephosphorylation of Atg13 and an increase in the affinity of this protein for Atg1. Atg1-Atg13 association induces autophosphorylation and activation of Atg1, promoting autophagy [27–30]. Recent evidence**s **indicate that Atg1-13 complex regulates recycling of Atg proteins such as Atg9 and Atg23 functioning at the autophagy organization site called PAS (for the preautophagosomal structure) [31].

### 2.2. Nucleation

While the origin of the lipid donor membranes in autophagy is still obscure, endoplasmic reticulum, Golgi, and a so far undetermined organelle called “the phagophore” were suggested as lipid providers to autophagosomes. Whatever is the origin, autophagosomal membranes are build up de novo as crescent-shaped structures in PAS. In yeast, PAS is a prominent structure next to the vacuole, but in higher eukaryotes, several sites are involved. Nucleation of autophagosomes is initiated by a protein complex including Vps34, a class III phosphatidylinositol 3-OH kinase (PI3K), and Atg6/Vps30 (Beclin1 in mammals). Together with other regulatory proteins such as UVRAG (UV radiation Resistance Associated Gene), Bif-1, and Ambra, Atg6-containing complex plays a role in the regulation of Vps34 activity. PI3K activity of Vps34 leads to the accumulation of phosphatidylinositol 3-phosphate (PI3P). PI3P produced by Vps34 serves as a landing pad on PAS for proteins involved in autophagosome formation such as Atg18 and Atg2 [16, 32, 33].

### 2.3. Vesicle Expansion and Completion

Two ubiquitination-like conjugation systems play a role in autophagosome biogenesis. In the first reaction, Atg12 is conjugated to Atg5 in a covalent manner [34]. The conjugation reaction starts with the activation of Atg12 by an ubiquitin-activating enzyme (E1)-like protein Atg7. Atg12 is then transferred to Atg10, an ubiquitin-conjugating-like enzyme (E2)-like protein [35, 36]. Finally, Atg12 is covalently conjugated to Atg5. The conjugation allows the formation and stabilization of a larger complex containing Atg12, Atg5, and Atg16 [37]. This protein complex is necessary for the second ubiquitination-like reaction to occur and to allow autophagosome membrane elongation. Atg12/5/16 complex localizes to the outer membrane of the forming autophagosome, and, dissociates from it as soon as the vesicle is completed, underlining the fact that its role is regulatory rather than structural [38]. 

The second ubiquitination-like reaction involves Atg8 protein (microtubule-associated protein light chain-3 or shortly LC3 in mammals). E1-like protein Atg7 activates Atg8 and transfers it to Atg3. While Atg7 is common to both conjugation reactions, E2-like protein Atg3 is specific for Atg8 conjugation to a lipid molecule (phosphatidylethanolamine, PE) [39]. Prior to conjugation, Atg8 has to be cleaved at its carboxy-terminus by Atg4, allowing the access of the lipid molecule to a Glycine residue on Atg8. Lipidation reaction is reversible since Atg4 can also cleave the conjugated lipid, enabling recycling of Atg8. Recent data provide evidence that together with Atg3, Atg12/5 complex is directly responsible for Atg8-PE conjugation [40]. The yeast Atg8 has several orthologues and isoforms in plants [41–43]. In the model plant *Arabidopsis thaliana*, at least 9 Atg8 proteins were described [44].

### 2.4. Autophagosome/Lysosome Fusion and Degradation

Autophagosomes fuse with late endosomes or lysosomes to form autolysosomes. Specific factors have been implicated in this step. A Vps complex and Rab GTPases proteins are involved in the organization of the fusion site. Then, SNAREs proteins (SNAP as soluble NSF attachment protein receptor) [45] form a complex which serves as a bridge between the two organelles [46, 47].

### 2.5. Recycling

In the lumen of lysosome/vacuole, lipases such as Atg15 first degrade the remaining autophagic membrane and the cargo is then catabolized by lysosomal lytic enzymes [48]. Following the degradation of the vesicle, building blocks are carried to cytosol for further use. Specialized lysosome membrane proteins play a role in this process including lysosomal-associated membrane proteins LAMP-1 and LAMP-2.

## 3. Plant Autophagy

Both microautophagy and macroautophagy are functional in plants [5]. Mechanisms of these pathways are similar to those described in other model organisms. 

In plant microautophagy, the target material is directly engulfed by an invagination of the tonoplast. Cargo-containing vesicle pinches off to be released inside the vacuole and degraded within the lumen. Microautophagy was involved in accumulation of storage proteins, lipids, and degradation of starch granules in developing plants [49, 50].

As in other organisms, the macroautophagy (hereafter “autophagy”) in plants is a process that starts with the formation of cup-shaped membranes in the cytoplasm. After completion, autophagosomes have at least two destinations in plants. They may fuse with the tonoplast and be directly delivered to the lumen of the vacuole as seen in *Arabidopsis*. Alternatively, autophagosomes may first transform into lysosome-like acidic and lytic structures and, fusion with the central vacuole may occur as a secondary event (Figures 1(b) and 1(c)) [51, 52].

In the model plant *Arabidopsis thaliana*, 25 orthologs of 12 yeast *ATG* genes were identified [44, 53–55]. Some exist as a single copy (i.e., Atg3 and Atg5) and others as multiple copies (i.e., Atg1 and Atg8). Functional domains of these *Arabidopsis* proteins were well conserved during evolution, indicating preservation of basic autophagy mechanisms in plants. Indeed, complementation tests in *ATG* mutant yeast strains using some of the plant Atg proteins confirmed the preservation of their function [43]. Moreover, gene targeting studies in whole plants demonstrated that plant genes of all tested autophagy proteins (i.e., for Atg7, Atg9 and Atg5-Atg12) were necessary for autophagosome formation following various types of stress [44, 53, 55]. Furthermore, some *ATG* genes were upregulated under stress conditions stimulating autophagy [7, 56–61]. A list of *Atg* genes identified in *Arabidopsis* and the phenotypes caused by their modification are depicted in Table 1.

### 3.1. Basal Autophagy in Plants

Autophagy is constitutively active in plant cells as in other organisms. Indeed, incubation of root tips with vacuolar enzyme inhibitors led to the accumulation of autophagic vesicles as autolysosome-like structures and in the vacuole. When cysteine protease inhibitor, E64d, was used to inhibit autophagy, autophagic vesicles accumulated inside vacuoles in *Arabidopsis* cells [13]. Similarly, growth of tobacco cells in the presence of E64d led to the accumulation of autolysosome-like structures outside the vacuole [52]. Autophagy-specific inhibitor 3-MA blocked the accumulation of autophagosomes and autolysosomes, demonstrating that autophagy is responsible for vesicle accumulation [52, 62]. Expression of a GFP fusion construct of *Atg8f* (an autophagy marker in *Arabidopsis*) resulted in the accumulation of this marker protein in the vacuole lumen. Atg8f accumulation was also detected in the presence of concanamycin A (a Vacuolar H(+)-ATPase inhibitor blocking vacuolar degradation) [57]. 

The role of constitutive autophagy in the degradation of damaged or oxidized molecules was confirmed using mutants of *AtAtg18a*. These mutants produced greater amounts of oxidized proteins and lipids in comparison to wild-type plants. Increased amount of oxidized protein and lipid generation in *Atg18a-*silenced plants underlined importance of autophagy for the degradation of oxidized molecules in plant cells [8, 63]. Therefore, as in other organisms, plant basal autophagy seems to function to eliminate damaged organelles (e.g., chloroplast, a source of reactive oxygen species in plants) and to clear damaged/abnormal proteins that accumulate in the cytoplasm [64].

### 3.2. Autophagy in Plant Development

The role of autophagy for plant development was studied using several autophagy gene mutants. Under nutrient-rich conditions, autophagy-defective plants achieve normal embryonic development, germination, shoot and root growth, flower development, and seed generation [44, 53, 54]. When these plants are grown under carbon- or nitrogen-deficient conditions, accelerated bolting, increased chlorosis, dark-induced senescence, and a decrease in seed yield were observed. Therefore, autophagy seems to be a major mechanism of nutrient mobilization under starvation conditions in plants.

Autophagy plays a role during vacuole biogenesis as well. In a recent study, Yano et al. [65] proposed that formation of vacuoles from tobacco BY-2 protoplasts involved an autophagy-like process. However, this process could not be inhibited by classical autophagy inhibitors such as 3-MA and wortmannin, suggesting that autophagy during vacuole formation differs from constitutive autophagy taking place under normal conditions or autophagy induced by stress.

### 3.3. Autophagy, Stress, and Cell Death

When organisms including plants are exposed to adverse environmental conditions, they develop responses to cope with stress and to survive. One of the major processes exploited by plant cells for this purpose is autophagy. Stress conditions inducing autophagy include sucrose, nitrogen, and carbon starvation, as well as oxidative stress and pathogen infection [8, 62, 66, 67]. For example, sucrose starvation has been reported to induce autophagy in rice [68], sycamore [6], and tobacco-cultured cells [69], and carbon starvation induced autophagy in maize plants [70]. Furthermore, autophagy participates in the formation of protein storage vacuoles in seeds and cereal grains [71, 72], prolamin internalization to vacuole in wheat [73], biogenesis of vegetative vacuoles in mature meristematic cells [74, 75], and degradation of proteins in protein storage vacuoles in mung bean [49, 76].

Since plants have a rigid cell wall and they lack typical caspase proteases, apoptosis is not the mechanism utilized by plants to degrade cellular components before cell death. During programmed cell death (PCD) in plants, vacuole and cell size increase, organelles are taken up by vacuole and subsequently degraded, and finally vacuole lyses resulting in cell death. These events overlap with the major characteristics of autophagy in plants [15, 77]. In the light of these observations, the role of autophagy in plant programmed cell death needs to be further investigated.

To avoid spread of infection, plants developed an innate immune response, called the hypersensitive response programmed cell death (HR-PCD). The innate immunity is achieved through limitation of the infection with the death of cells surrounding the infected area [78]. Studies using autophagy gene mutant plants showed that an autophagy defect is associated with a failure to contain cell death at the infection site, leading to its spread into uninfected tissue [79–81]. Therefore, paradoxically, autophagy also plays a role in limiting cell death initiated during plant innate immune responses. Indeed, as seen in plants, autophagy is involved both in cell survival and cell death in various other organisms [12].

## 4. Techniques to Study Autophagy

Various techniques and tools were used to monitor and evaluate autophagy. While transmission electron microscopy (TEM) analysis remains “the golden standard,” with the recent advances in the field, several new molecular tools are being introduced. The possibility of their usage in plant autophagy research will be discussed.

### 4.1. Electron Microscopy

**Figure 2:**
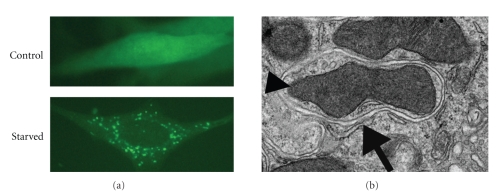
GFP-Atg8/LC3 dot accumulation and TEM method to detect autophagic activity. (a) LC3 dot formation upon starvation in fibroblasts isolated from GFP-Atg8/LC3 transgenic mice. The green dots are autophagic vesicles labelled by GFP-Atg8/LC3. (b) Transmission electron microscopic picture of an autophagic vesicle (arrow) in kidney of tunicamycin injected mouse. Note that in addition to cytoplasmic material, a mitochondrium (arrowhead) is also engulfed inside the double membrane vesicle.

Transmission electron microscopy (TEM) is one of the earliest tools used to characterize autophagy [82], and it is still one of the most reliable methods to monitor autophagy in cells and tissues. Yet, interpretation of the TEM data requires special expertise and there are several criteria to describe autophagosomes and autolysosomes with precision. The hallmark of autophagosomes is their double or multimembrane structures containing electron dense material with a density similar to that of the cytoplasm. Presence in autophagosomes of organelles such as mitochondria, chloroplasts, and endoplasmic reticulum (ER) strengthens the conclusion (Figure 2(b)). Autolysosomes contain darker, degenerated, or degraded material and some of them are reminiscent of lysosomes/vacuole. 

Other cytoplasmic figures may be erroneously described as autophagosomes and autolysosomes. Degenerated mitochondria, folds of ER, or nuclear membrane may be mistaken for autophagosomes [83–85]. Sometimes the typical double membrane structure of autophagosomes may be disrupted (e.g., following infection with some pathogens) [86]. Therefore, unbiased and clear identification of autophagosomes using TEM requires extreme precaution. Combination of electron microscopy with immunogold-labelling of autophagosome-specific markers such as Atg8/LC3 may allow a more objective and reliable interpretation depending on the experimental needs [87]. Transmission electron microscopy was successfully used to detect autophagy in plants [61, 79].

### 4.2. Molecular Markers

Proteins that are involved in the autophagy process or that are degraded specifically through autophagy have been used to monitor autophagic activity. Several of them are already in use in plants. Plants knock-out and transgenic for these markers are useful tools to study autophagy-related phenotypes under different experimental conditions (see Table 1). Molecular techniques, such as Atg8/LC3 dot formation, were successfully used for high-throughput screens of autophagy in various systems [88]. 

#### 4.2.1. Atg8/LC3 Dot Formation and Accumulation of Its Lipidated Form

Atg8/LC3 is covalently conjugated to a lipid molecule as a result of an ubiquitination-like reaction and, its lipidation is required for autophagic membrane elongation (see Section 2.3). In plants, several isoforms of Atg8/LC3 seem to be functional during autophagy mechanisms [57]. During autophagy, Atg8/LC3 lipidation and recruitment to autophagic membranes changes its localization from diffuse cytosolic to punctuate (Figure 2) [51, 54, 89, 90]. Moreover, in SDS-PAGE protein gels, the molecular weight of Atg8/LC3 changes from 18kDa (free cytosolic form, free Atg8, or LC3-I) to 16kDa (lipidated form, Atg8-PE (or LC3-II)) [41, 54, 57]. Soon after the discovery of its autophagy-related lipidation, Atg8/LC3 had become one of the main tools to monitor autophagy. The localization change of an Atg8/LC3-fluorescent protein fusion construct (such as GFP-Atg8/LC3) is commonly used to detect autophagy in cells (Figure 2(a)) and in whole organisms including transgenic *Arabidopsis* and tobacco plants [38, 51, 54, 55, 57].

When working with isolated cells, quantification of GFP-Atg8/LC3 signal using FACscan/flow cytometer may be used as an autophagy evaluation tool [91]. In this system, induction of autophagy led to a decrease in GFP-Atg8/LC3 signal. Conversely the fluorescent signal increased following the usage of autophagy inhibitors. This method is a good quantitative tool to monitor activity in living cells by FACscan/flow cytometer [92–94], especially using cells derived from Atg8 transgenic plants. 

Nevertheless some precautions must be taken even when using this popular molecular marker. Free Atg8 (or LC3-I) to Atg8-PE (or LC3-II) ratio differ among tissues, depending on stimuli and antibodies that are used, therefore, reliable controls must be added [95]. To avoid misinterpretations due to kinetics of autophagy, it is highly advised to check Atg8/LC3 lipidation at several time points after signal application rather than using only one point in time [95]. The use of vacuolar/lysosomal degradation inhibitors will help to confirm that accumulation of the lipidated form is indeed due to the canonical autophagy pathway. 

Atg8/LC3 lipidation and cytosolic dot formation may not always reflect activation of autophagy. It has been reported that high level GFP-Atg8/LC3 expression may also lead to dot formation even in nonautophagic cells [96] and in autophagy mutants [97]. Furthermore, Atg8/LC3 was found to associate with protein aggregates marked with p62/SQSTM1 (see Section 4.2.7) in an autophagy-independent manner [98]. Importantly, Atg8/LC3 lipidation reflects an early stage in autophagosome formation and it cannot be interpreted as autophagic activity *per se* [99, 100]. Hence, this method should not be used as the only technique to monitor autophagy and it has to be complemented with other autophagy detection techniques including TEM analysis [95].

#### 4.2.2. Atg6 and Phosphatidyl Inositol 3-Phosphate Detection

The role of Atg6 in autophagy has been extensively studied. As stated before, Atg6 regulates Vps34 class III phosphoinositide-3 kinase (PI3K) complex producing PI3P that is involved in autophagic vesicle nucleation. Similar to Atg8/LC3, intracellular localization change of a fluorescent protein fusion of Atg6 (and leading to its colocalization with PI3P) was observed upon autophagy induction [101, 102]. PI3P may be labelled in cells using a PI3P-binding peptide, FYVE fused to GFP [103]. Quantification of the accumulation of GFP-FYVE-labelled dots may also be used as a tool to quantify autophagy activation upon starvation in mammalian cells (Yamaner Y. and Gozuacik D. unpublished data). Adaptations to the plant system may be possible since orthologues of Atg6 and Vps34 are present in plants including *Arabidopsis* [104].

#### 4.2.3. Atg5 and Atg16

Atg5 as well as Atg16 was used as a selective marker to recognize autophagosome organization centers (PAS). Since Atg5 dissociates after vesicle completion, it will not label autophagosomes or lysosomes. The signal could be detected as fluorescent dots under microscope [38, 97]. A recent study used Atg16L as a new marker to detect autophagosome formation [105]. Like Atg5, Atg16L transiently associates with the surface of autophagosomes during their formation and forms punctate structures [106]. Therefore, as Atg8/LC3, Atg5 and Atg16L, coupled with a fluorophore or detected by immunofluorescence using specific antibodies, can be used to monitor autophagosome formation. As homologues of Atg5 and Atg16 exist in plants (e.g., *Arabidopsis, Z. mays*) this technique might be useful in plants studies as well.

#### 4.2.4. Atg18

A mammalian orthologue of the yeast Atg18, WIPI-1, was proposed as a marker for autophagy as well [107]. WIPI-1 is a WD (Tryptophan and aspartic acid) repeat protein [108] and as such, it may interact with PI3P and accumulate in dot-like structures (upon autophagy induction by amino acid starvation other stimuli). WIPI-1 dots were shown to colocalize with Atg8/LC3 [107, 109] in human cells lines. Whether plant Atg18 protein might be used as an autophagy marker has to be tested as homologues are found in plants such as *Arabidopsis*.

#### 4.2.5. Atg4 Activity

Cleavage of Atg8/LC3 by Atg4 cysteine protease is a crucial step before its lipidation. Recently, monitoring Atg8/LC3 cleavage by Atg4 was proposed as a technique to detect autophagy [110]. The assay is based on the cleavage by Atg4 of a luciferase protein fused to Atg8/LC3 which, itself, is fixed on actin cytoskeleton. In this system, actin-associated luciferase has a secretion signal and, upon cleavage of Atg8/LC3 by Atg4, it is released from the cell. Luciferase activity can then be quantified in cellular supernatants reflecting Atg4 activity. Free luciferase can also be visualized in protein blots. Homologues of Atg4 are present in plants including *Arabidopsis* and rice; therefore, this technique could be adapted to monitor Atg4 protease activity in plants.

#### 4.2.6. Atg1 Activity

Atg1 is a serine/threonine kinase. Its activity correlated with autophagy induction [22, 27, 111–113]. In *S. cerevisiae*, Atg1 autophosphorylation is dramatically reduced upon starvation leading to autophagy [28]. In mammals, the function of Atg1 orthologues Ulk1 and Ulk2 seems to be controlled by autophosphorylation as well [113, 114]. Hence, Atg1 kinase activity and phosphorylation status could be used as a new test of the autophagic activity in cells, tissues, and extracts. In *Arabidopsis thaliana* genome, orthologues of the yeast genes coding for Atg1 kinase and Atg13 have been identified [53, 115]. Therefore, measuring Atg1 activity could serve as a tool to monitor autophagy in plants.

#### 4.2.7. p62/SQSTM1

Sequestosome 1 (SQSTM1), also named ubiquitin-binding protein p62 (shortly p62), is a stress-induced adaptor/marker protein that is a common component of protein aggregates [116]. p62 was shown to bind Atg8/LC3 proteins through its N-terminal region [117]. p62/Atg8 interaction triggered degradation of protein aggregates by autophagy during which p62 itself was also degraded [118, 119]. This observation led to the use of p62 degradation as a molecular tool to detect autophagic activity [119–121]. As LC3 lipidation appears prior to p62 degradation, existence of a lag phase should be considered during the design of the experiments [95]. Of note, it is still not known whether p62 is a general marker for autophagy and caution should be taken when using this technique with new autophagy-inducing stimuli. Our preliminary analyses revealed that there are no p62 orthologues in *Arabidopsis*. Yet, we cannot exclude the possibility that p62-like proteins exist in plants.

### 4.3. Tests of Lysosomal/Vacuolar Activity

#### 4.3.1. Lysotracker

Weakly basic amines selectively accumulate in cellular compartments with low internal pH and can be used to visualize acidic compartments such as lysosomes/vacuoles. Lysotracker is a fluorescent acidotropic probe used for labeling acidic organelles in live cells. It consists of a fluorophore linked to a weak base. Labelling of acidic compartments by lysotracker is likely due to its protonation and retention in the membranes of these organelles. Lytic compartment labelling methods such as lysotracker staining must be used in combination with more specific markers of autophagy in order to discriminate autophagic activity from other events increasing lysosome/vacuole activity. Lysotracker staining method has been used to monitor autophagy in various organisms including *Arabidopsis*, tobacco, and barley [79, 80, 122].

#### 4.3.2. Acridine Orange (AO)

AO is a fluorescent basic dye that has the ability to cross biological membranes. AO accumulates in acidic compartments, such as lysosomes and vacuole, and becomes protonated and sequestered in their lumen. In acridine orange-stained cells, cytoplasm and nucleolus emit bright green fluorescence, whereas acidic compartments fluoresce in bright red. Therefore, quantification of the red fluorescence reflects the degree of acidity and the volume of the cellular acidic compartments. Comparison of the ratio of green/red fluorescence in cells, using fluorescent microscopy or flow cytometry, enables quantification of the extent of autophagic degradation [123, 124]. So far, to our knowledge, no study used AO as a plant autophagy marker.

#### 4.3.3. Monodansylcadaverine (MDC)

The autofluorescent substance monodansylcadaverine is commonly used to detect autophagy in plants and in other organisms [67, 125–127]. MDC is a weak base that is capable of crossing biological membranes and concentrating in acidic compartments [128]. Although MDC was originally proposed to label autophagosomes and autolysosomes, recent studies on mammalian autophagy brought out that it is not an autophagy-specific marker. These publications revealed that MDC-positive structures colocalized only partially with autophagosome markers in cells [129]. Furthermore, in autophagy-defective Atg5 knockout cells, MDC-positive dots were still observed [130]. The figures labelled by MDC seem to be endosomes, lysosomes, and lamellar bodies [125]. Therefore, MDC associates with acidic and lipid-rich compartments and it does not discriminate between autophagosomes/autolysosomes and the aforementioned vesicular organelles. Hence, MDC staining has to be combined with other techniques to avoid misinterpretations. Whether MDC is also labelling nonautophagic structures in plants needs careful investigation.

### 4.4. Biochemical Methods

#### 4.4.1. Long-Lived Protein Degradation

Since autophagy is involved in the degradation of long-lived proteins, determination of their turnover appears to be an efficient method to monitor autophagy levels in cells. In the commonly used technique, following metabolic labelling, degradation of all long-lived proteins is measured. A radioactively labelled amino acid such as valine or leucine can be used to label newly synthesized proteins. Then cells are incubated with cold amino acids to allow short-lived proteins to be degraded. Finally, release of labelled amino acids resulting from the degradation of long-lived proteins is monitored [131]. 

One major weakness of this technique is that autophagy is not the only mechanism of long-lived proteins degradation. Autophagic and nonautophagic degradation of long-lived proteins should be distinguished by the use of autophagy inhibitors such as 3-mehyladenine (3-MA) [132]. An alternative nonradioactive method uses chromatography to monitor the amount of released unlabeled amino acids [133]. 

Usage of metabolic labelling in plants was hindered by high compartmentalization of protein substrates and by the fact that metabolite pools in plant cells are generally highly dynamic [134]. Recently developed techniques allowing metabolic labeling of whole plants and plant cell cultures may overcome these difficulties and allow quantification of autophagy by long-lived protein degradation in plants [135–137].

#### 4.4.2. Sequestration of Sugars

Radio-labelled sucrose or raffinose, delivered to cytosol through electropermeabilization, is sequestered in autophagic vesicles together with engulfed cytosolic fragments. Accumulation of radioactivity in autophagic membrane fractions was used to measure autophagic activity [138, 139]. This method has its limitations as well. For example, it cannot be used in yeast due to fast metabolism [140]. Furthermore, injection of the labelled molecule can disturb cellular homeostasis, therefore, precautions and extracontrols including determination of the metabolic equilibrium of the cell prior to the measurement are required. Sugar sequestration technique might be useful in plant cell cultures studies and it needs to be tested.

#### 4.4.3. Phosphorylcholine Accumulation

An assay to monitor autophagy in plants is based on the followup of phosphorylcholine accumulation in cells. The technique was developed in sycamore suspension cells cultures undergoing autophagy upon sucrose starvation [6]. Carbon starvation-activated degradation of membrane lipids led to the accumulation of phosphorylcholine in the cytoplasm. Phosphorylcholine accumulation correlated well with autophagy-induction and its quantification by 31P-NMR spectroscopy was proposed as a novel way of autophagy detection in plant cells.

### 4.5. Other Techniques

#### 4.5.1. Nonselective Degradation of Cytosolic Proteins

One of the yeast techniques developed to monitor autophagy makes use of an N-terminal truncated mutant of the yeast alkaline phosphatase Pho8 [141]. In contrast to the ER-localized wild-type enzyme, the mutant form of pho8 lacking the N-terminal signal sequence (Pho8*δ*60), is delivered to the vacuole by way of autophagy. Following entry to the vacuole, Pho8*δ*60 is cleaved at its C-terminus to produce the active alkaline phosphatase. Measurement of alkaline phosphatase activity and/or protein immunoblotting to check the shift between precursor and mature enzyme allows the detection of autophagic activity in yeast cells. Nonselective degradation of marker proteins (especially those with an enzymatic activity) might also be used in plants as autophagy detection methods.

#### 4.5.2. Selective Autophagic Degradation of Proteins

Although autophagy is generally considered as a nonselective phenomenon, some proteins appear to be selectively degraded by autophagy. A GFP or DsRed construct, targeted to the chloroplast, and a GFP fusion of rubisco were transported to the vacuole through autophagy [90, 142]. Rubisco is allocated most of the plant nitrogen and functions in carbon-fixation in chloroplasts. It is released from the chloroplasts in structures called rubisco-containing bodies (RCBs) in order to provide nitrogen from the leaves to others organs. RCB seem to overlap with autophagic vesicles, indicating that rubisco is engulfed in autophagosomes and eventually delivered to the vacuole. The process was dependent on *ATG* genes underlining the autophagic character of the transport. Therefore, targeted GFP-DsRed constructs or GFP-Rubisco may be used as tools to study selective autophagy in plants. 

Another specific target of autophagy is betaine homocysteine methyltransferase. Accumulation of this protein in autophagosomes and its cleavage in the lysosome was observed [143]. Another study proposed measurement of neomycin phosphotransferase II accumulation by flow cytometry as an autophagy detection method [144, 145]. Whether the plant orthologue betaine homocysteine methyltransferase shares the same faith and whether neomycin phosphotransferase follows the same path in plants has to be determined.

#### 4.5.3. Tests of Mitochondrial Autophagy (Mitophagy)

Since autophagy is a general process for the quality control of organelles, mitochondria are common targets of autophagic degradation. The term mitophagy was coined to describe the selective degradation of mitochondria by autophagy [146]. In yeast, a technique of mitophagy detection was recently developed. This method is based on the use of a GFP-tagged mitochondrial protein and monitorization of the vacuolar release of green fluorescent protein after the degradation of chimera [147]. Indeed, degradation of mitochondrial proteins was previously used to monitor autophagy [148]. Similarly, during autophagy activated by sucrose starvation in plants, a gradual decrease in the number of mitochondria per cell was observed, indicating that techniques based on mitochondrial degradation may be developed to study autophagy in plants [149].

## 5. Concluding Remarks

Due to its role in fundamental biological phenomena in various organisms including humans and plants, interest in autophagy field is growing exponentially [150]. Accumulation of the knowledge on autophagy molecular mechanisms stimulated the discovery of more efficient and reliable molecular tools to study autophagy. Despite the fact that some of these methods and tools seem to be more suitable for use in specific model organisms, adaptations should be possible in many cases. Plant autophagy studies already benefit from the adaptation of various general autophagy detection techniques used in other model organisms, such as Atg8/LC3 localization tests. Main disadvantages or difficulties of available tools to study autophagy are depicted in Table 2. A better understanding of the biological phenomena involving autophagy in plants and its molecular mechanisms and targets will lead to the development of novel and more precise techniques that will allow the measurement of autophagy in plants with increasing precision and will further accelerate studies in this field.

## Figures and Tables

**Table 1:** Phenotypes caused by ATG gene modifications in *Arabidopsis thaliana*. E64d, inhibitor of lysosomal/vacuolar hydrolases; Concanamycin A, inhibitor of vacuolar (V-type) ATPase, preventing lysosomal/vacuolar degradation:HR-PCD (hypersensitive response programmed cell death).

Genotype	Phenotype	Reference(s)
Atg2-deficient	No autophagic inclusions in root tips upon E64d treatment.	[52]
Atg4a-/ Atg4b-deficient	Upon nitrogen starvation, no autophagosome formation and no delivery of GFP-Atg8 to the vacuole.	[54]
Atg5-deficient	Inhibition of rubisco containing body formation.	[90]
	No autophagic vesicles in root tips after E64d treatment.	[52]
	No formation of Atg5/12 complex. Defective in autophagy induced by concanamycin A treatment.	[151]
	Senescence upon light and carbon or nitrogen limitation.	[55]
Atg6-deficient	Male sterility.	[152]
	HR-PCD sensitive. Early senescence.	[80]
	Developmental defects and impaired pollen germination.	[153]
Atg7-deficient	Hypersensitive to nutrient-limitation. Senescence.	[44]
Atg8-transgenic	Expression induced by starvation. Stress leads premature aging.	[57, 66]
Atg9-deficient	Under carbon and nitrogen starvation, accelerated chlorosis.	[53]
	Seed germination impaired and leaf senescence accelerated.	
	Weak decrease of autophagic vesicle accumulation following E64d treatment.	[52]
Atg10-deficient	Hypersensitive to nitrogen and carbon starvation. Early senescence and PCD.	[89]
	No formation of Atg5/12 complex. Defective in autophagy induced by concanamycin A treatment.	[151]
Atg18a-transgenic	Hypersensitivity to sucrose and nitrogen starvation. Premature senescence.	[154]

**Table 2:** Advantages and disadvantages of techniques used to study autophagy.

Technique	Advantages	Disadvantages
Electron microscopy	Golden standard.Morphological characterization of autophagosomes, autolysosomes and their cargo.	Equipment and expertise required.Difficult to make quantitative analyses.
Atg8/LC3 conjugation to lipid	Rapid detection and quantification of autophagy.Amenable to high throughput techniques.Used to create transgenic organisms for in vivo study of autophagy.	Dots do not always reflect autophagic activity.Molecular weight shift tests need careful interpretation.
Other molecular markers (Atg5, Atg6, Atg16 and Atg18 detection)	Detection of various stages of autophagic vesicle formation.	Most of them need further evaluation.
PI3P detection	Reflects the activity of Vps34 kinase.Quantitative analysis possible.	PI3P accumulation in phenomena not directly related to autophagy (vesicular transport).
Atg1 and Atg4 activity	Determination of enzymatic activity.	So far no clear kinetic studies were published.
P62/SQSTM1 degradation	Activated especially by protein aggregates.	Not all stimuli activate its degradation.Orthologue in plants?
Lysotracker and acridine orange staining	Determination and quantification of autophagy-related lytic activity (lysosomal/vacuolar).FACscan analysis possible.	Autophagosomes are not detected.Lytic activity induced by other conditions as well.
MDC staining	Determination and quantification of autophagy-related lytic activity (lysosomal/vacuolar).	Not all autophagosomes are detected.Lytic activity induced by other conditions as well.
Long-lived protein degradation	Measures autophagic degradation of proteins.Kinetic measurements possible.	Nonspecific degradation of proteins by mechanisms other than autophagy. Radioactive technique.
Sequestration of sugars	Measures autophagic sequestration phase.Quantification may be possible.	Sugars may be metabolized.
Phosphorylcholine accumulation	Promising plant autophagy technique.Quantification may be possible.	Quantification requires special equipment (NMR spectroscopy).
Nonselective and selective degradation of proteins	Promising techniques for plant autophagy.Detection of both sequestration and degradation phases.Quantification may be possible.	Autophagy target proteins need further characterization.
Test of mitophagy or chloroplast autophagy	Detection of autophagy target organelle degradation.Various organelle-specific proteins or organelle-tagged may be used.	Quantification not always possible.

## References

[1] Dunn WA, Cregg JM, Kiel JA (2005). Pexophagy: the selective autophagy of peroxisomes. *Autophagy*.

[2] Mortimore GE, Lardeux BR, Adams CE (1988). Regulation of microautophagy and basal protein turnover in rat liver. Effects of short-term starvation. *The Journal of Biological Chemistry*.

[3] Klionsky DJ (2005). The molecular machinery of autophagy: unanswered questions. *Journal of Cell Science*.

[4] Massey AC, Zhang C, Cuervo AM (2006). Chaperone-mediated autophagy in aging and disease. *Current Topics in Developmental Biology*.

[5] Bassham DC, Laporte M, Marty F (2006). Autophagy in development and stress responses of plants. *Autophagy*.

[6] Aubert S, Gout E, Bligny R (1996). Ultrastructural and biochemical characterization of autophagy in higher plant cells subjected to carbon deprivation: control by the supply of mitochondria with respiratory substrates. *The Journal of Cell Biology*.

[7] Rose TL, Bonneau L, Der C, Marty-Mazars D, Marty F (2006). Starvation-induced expression of autophagy-related genes in *Arabidopsis*. *Biology of the Cell*.

[8] Xiong Y, Contento AL, Nguyen PQ, Bassham DC (2007). Degradation of oxidized proteins by autophagy during oxidative stress in *Arabidopsis*. *Plant Physiology*.

[9] Thompson AR, Vierstra RD (2005). Autophagic recycling: lessons from yeast help define the process in plants. *Current Opinion in Plant Biology*.

[10] Seay M, Patel S, Dinesh-Kumar SP (2006). Autophagy and plant innate immunity. *Cellular Microbiology*.

[11] Gutierrez MG, Master SS, Singh SB, Taylor GA, Colombo MI, Deretic V (2004). Autophagy is a defense mechanism inhibiting BCG and *Mycobacterium tuberculosis* survival in infected macrophages. *Cell*.

[12] Gozuacik D, Kimchi A (2007). Autophagy and cell death. *Current Topics in Developmental Biology*.

[13] Bassham DC (2007). Plant autophagy—more than a starvation response. *Current Opinion in Plant Biology*.

[14] van Doorn WG, Woltering EJ (2005). Many ways to exit? Cell death categories in plants. *Trends in Plant Science*.

[15] Horner HT, Healy RA, Cervantes-Martinez T, Palmer RC (2003). Floral nectary fine structure and development in *Glycine max* L. (Fabaceae). *International Journal of Plant Sciences*.

[16] Xie Z, Klionsky DJ (2007). Autophagosome formation: core machinery and adaptations. *Nature Cell Biology*.

[17] Klionsky DJ, Cregg JM, Dunn WA (2003). A unified nomenclature for yeast autophagy-related genes. *Developmental Cell*.

[18] Gozuacik D, Kimchi A (2004). Autophagy as a cell death and tumor suppressor mechanism. *Oncogene*.

[19] Thomas G, Hall MN (1997). TOR signalling and control of cell growth. *Current Opinion in Cell Biology*.

[20] Dann SG, Thomas G (2006). The amino acid sensitive TOR pathway from yeast to mammals. *FEBS Letters*.

[21] Díaz-Troya S, Pérez-Pérez ME, Florencio FJ, Crespo JL (2008). The role of TOR in autophagy regulation from yeast to plants and mammals. *Autophagy*.

[22] Noda T, Ohsumi Y (1998). Tor, a phosphatidylinositol kinase homologue, controls autophagy in yeast. *The Journal of Biological Chemistry*.

[23] Kunz J, Henriquez R, Schneider U, Deuter-Reinhard M, Movva NR, Hall MN (1993). Target of rapamycin in yeast, TOR2, is an essential phosphatidylinositol kinase homolog required for G_1_ progression. *Cell*.

[24] Sormani R, Lei Y, Menand B (2007). *Saccharomyces cerevisiae* FKBP12 binds *Arabidopsis thaliana* TOR and its expression in plants leads to rapamycin susceptibility. *BMC Plant Biology*.

[25] Beck T, Hall MN (1999). The TOR signalling pathway controls nuclear localization of nutrient-regulated transcription factors. *Nature*.

[26] Natarajan K, Meyer MR, Jackson BM (2001). Transcriptional profiling shows that Gcn4p is a master regulator of gene expression during amino acid starvation in yeast. *Molecular and Cellular Biology*.

[27] Kamada Y, Funakoshi T, Shintani T, Nagano K, Ohsumi M, Ohsumi Y (2000). Tor-mediated induction of autophagy via an Apg1 protein kinase complex. *The Journal of Cell Biology*.

[28] Matsuura A, Tsukada M, Wada Y, Ohsumi Y (1997). Apg1p, a novel protein kinase required for the autophagic process in *Saccharomyces cerevisiae*. *Gene*.

[29] Abeliovich H, Zhang C, Dunn WA, Shokat KM, Klionsky DJ (2003). Chemical genetic analysis of Apg1 reveals a non-kinase role in the induction of autophagy. *Molecular Biology of the Cell*.

[30] Codogno P (2004). [ATG genes and macroautophagy]. *Médecine Sciences*.

[31] Reggiori F, Tucker KA, Stromhaug PE, Klionsky DJ (2004). The Atg1-Atg13 complex regulates Atg9 and Atg23 retrieval transport from the pre-autophagosomal structure. *Developmental Cell*.

[32] Kihara A, Noda T, Ishihara N, Ohsumi Y (2001). Two distinct Vps34 phosphatidylinositol 3-kinase complexes function in autophagy and carboxypeptidase Y sorting in *Saccharomyces cerevisiae*. *The Journal of Cell Biology*.

[33] Petiot A, Ogier-Denis E, Blommaart EFC, Meijer AJ, Codogno P (2000). Distinct classes of phosphatidylinositol 3′-kinases are involved in signaling pathways that control macroautophagy in HT-29 cells. *The Journal of Biological Chemistry*.

[34] Mizushima N, Noda T, Yoshimori T (1998). A protein conjugation system essential for autophagy. *Nature*.

[35] Shintani T, Mizushima N, Ogawa Y, Matsuura A, Noda T, Ohsumi Y (1999). Apg10p, a novel protein-conjugating enzyme essential for autophagy in yeast. *The EMBO Journal*.

[36] Tanida I, Mizushima N, Kiyooka M (1999). Apg7p/Cvt2p: a novel protein-activating enzyme essential for autophagy. *Molecular Biology of the Cell*.

[37] Mizushima N, Noda T, Ohsumi Y (1999). Apg16p is required for the function of the Apg12p-Apg5p conjugate in the yeast autophagy pathway. *The EMBO Journal*.

[38] Mizushima N, Yamamoto A, Hatano M (2001). Dissection of autophagosome formation using Apg5-deficient mouse embryonic stem cells. *The Journal of Cell Biology*.

[39] Ichimura Y, Kirisako T, Takao T (2000). A ubiquitin-like system mediates protein lipidation. *Nature*.

[40] Fujioka Y, Noda NN, Fujii K, Yoshimoto K, Ohsumi Y, Inagaki F (2008). In vitro reconstitution of plant Atg8 and Atg12 conjugation systems essential for autophagy. *The Journal of Biological Chemistry*.

[41] Kabeya Y, Mizushima N, Ueno T (2000). LC3, a mammalian homologue of yeast Apg8p, is localized in autophagosome membranes after processing. *The EMBO Journal*.

[42] Sagiv Y, Legesse-Miller A, Porat A, Elazar Z (2000). GATE-16, a membrane transport modulator, interacts with NSF and the Golgi v-SNARE GOS-28. *The EMBO Journal*.

[43] Ketelaar T, Voss C, Dimmock SA, Thumm M, Hussey PJ (2004). *Arabidopsis* homologues of the autophagy protein Atg8 are a novel family of microtubule binding proteins. *FEBS Letters*.

[44] Doelling JH, Walker JM, Friedman EM, Thompson AR, Vierstra RD (2002). The APG8/12-activating enzyme APG7 is required for proper nutrient recycling and senescence in *Arabidopsis thaliana*. *The Journal of Biological Chemistry*.

[45] Darsow T, Rieder SE, Emr SD (1997). A multispecificity syntaxin homologue, Vam3p, essential for autophagic and biosynthetic protein transport to the vacuole. *The Journal of Cell Biology*.

[46] Ungermann C, Langosch D (2005). Functions of SNAREs in intracellular membrane fusion and lipid bilayer mixing. *Journal of Cell Science*.

[47] Abeliovich H, Klionsky DJ (2001). Autophagy in yeast: mechanistic insights and physiological function. *Microbiology and Molecular Biology Reviews*.

[48] Kim I, Rodriguez-Enriquez S, Lemasters JJ (2007). Selective degradation of mitochondria by mitophagy. *Archives of Biochemistry and Biophysics*.

[49] Van der Wilden W, Herman EM, Chrispeels MJ (1980). Protein bodies of mung bean cotyledons as autophagic organelles. *Proceedings of the National Academy of Sciences of the United States of America*.

[50] Poxleitner M, Rogers SW, Samuels AL, Browse J, Rogers JC (2006). A role for caleosin in degradation of oil-body storage lipid during seed germination. *The Plant Journal*.

[51] Toyooka K, Moriyasu Y, Goto Y, Takeuchi M, Fukuda H, Matsuoka K (2006). Protein aggregates are transported to vacuoles by a macroautophagic mechanism in nutrient-starved plant cells. *Autophagy*.

[52] Inoue Y, Suzuki T, Hattori M, Yoshimoto K, Ohsumi Y, Moriyasu Y (2006). AtATG genes, homologs of yeast autophagy genes, are involved in constitutive autophagy in *Arabidopsis* root tip cells. *Plant & Cell Physiology*.

[53] Hanaoka H, Noda T, Shirano Y (2002). Leaf senescence and starvation-induced chlorosis are accelerated by the disruption of an *Arabidopsis* autophagy gene. *Plant Physiology*.

[54] Yoshimoto K, Hanaoka H, Sato S (2004). Processing of ATG8s, ubiquitin-like proteins, and their deconjugation by ATG4s are essential for plant autophagy. *The Plant Cell*.

[55] Thompson AR, Doelling JH, Suttangkakul A, Vierstra RD (2005). Autophagic nutrient recycling in *Arabidopsis* directed by the ATG8 and ATG12 conjugation pathways. *Plant Physiology*.

[56] Contento AL, Kim S-J, Bassham DC (2004). Transcriptome profiling of the response of *Arabidopsis* suspension culture cells to Suc starvation. *Plant Physiology*.

[57] Sláviková S, Shy G, Yao Y (2005). The autophagy-associated Atg8 gene family operates both under favourable growth conditions and under starvation stresses in *Arabidopsis* plants. *Journal of Experimental Botany*.

[58] Van Der Graaff E, Schwacke R, Schneider A, Desimone M, Flügge UI, Kunze R (2006). Transcription analysis of *Arabidopsis* membrane transporters and hormone pathways during developmental and induced leaf senescence. *Plant Physiology*.

[59] Osuna D, Usadel B, Morcuende R (2007). Temporal responses of transcripts, enzyme activities and metabolites after adding sucrose to carbon-deprived *Arabidopsis* seedlings. *The Plant Journal*.

[60] Wagstaff C, Yang TJW, Stead AD, Buchanan-Wollaston V, Roberts JA (2009). A molecular and structural characterization of senescing *Arabidopsis* siliques and comparison of transcriptional profiles with senescing petals and leaves. *The Plant Journal*.

[61] Ghiglione HO, Gonzalez FG, Serrago R (2008). Autophagy regulated by day length determines the number of fertile florets in wheat. *The Plant Journal*.

[62] Takatsuka C, Inoue Y, Matsuoka K, Moriyasu Y (2004). 3-methyladenine inhibits autophagy in tobacco culture cells under sucrose starvation conditions. *Plant & Cell Physiology*.

[63] Xiong Y, Contento AL, Bassham DC (2007). Disruption ol autophagy results in constitutive oxidative stress in *Arabidopsis*. *Autophagy*.

[64] Niwa Y, Kato T, Tabata S (2004). Disposal of chloroplasts with abnormal function into the vacuole in *Arabidopsis thaliana* cotyledon cells. *Protoplasma*.

[65] Yano K, Hattori M, Moriyasu Y (2007). A novel type of autophagy occurs together with vacuole genesis in miniprotoplasts prepared from tobacco culture cells. *Autophagy*.

[66] Slavikova S, Ufaz S, Avin-Wittenberg T, Levanony H, Galili G (2008). An autophagy-associated Atg8 protein is involved in the responses of *Arabidopsis* seedlings to hormonal controls and abiotic stresses. *Journal of Experimental Botany*.

[67] Contento AL, Xiong Y, Bassham DC (2005). Visualization of autophagy in *Arabidopsis* using the fluorescent dye monodansylcadaverine and a GFP-AtATG8e fusion protein. *The Plant Journal*.

[68] Chen MH, Liu LF, Chen YR, Wu Hsin Kan, Yu SM (1994). Expression of *α*-amylase, carbohydrate metabolism, and autophagy in cultured rice cells is coordinately regulated by sugar nutrient. *The Plant Journal*.

[69] Moriyasu Y, Ohsumi Y (1996). Autophagy in tobacco suspension-cultured cells in response to sucrose starvation. *Plant Physiology*.

[70] Brouquisse R, Gaudillère JP, Raymond P (1998). Induction of a carbon-starvation-related proteolysis in whole maize plants submitted to light/dark cycles and to extended darkness. *Plant Physiology*.

[71] Robinson DG, Hinz G, Holstein SEH (1998). The molecular characterization of transport vesicles. *Plant Molecular Biology*.

[72] Galili G, Herman EM (1997). Protein bodies: storage vacuoles in seeds. *Advances in Botanical Research*.

[73] Levanony H, Rubin R, Altschuler Y, Galili G (1992). Evidence for a novel route of wheat storage proteins to vacuoles. *The Journal of Cell Biology*.

[74] Marty F (1978). Cytochemical studies on GERL, provacuoles, and vacuoles in root meristematic cells of *Euphorbia*. *Proceedings of the National Academy of Sciences of the United States of America*.

[75] Marty F (1999). Plant vacuoles. *The Plant Cell*.

[76] Toyooka K, Okamoto T, Minamikawa T (2001). Cotyledon cells of *Vigna mungo* seedlings use at least two distinct autophagic machineries for degradation of starch granules and cellular components. *The Journal of Cell Biology*.

[77] Gaffal KP, Friedrichs GJ, El-Gammal S (2007). Ultrastructural evidence for a dual function of the phloem and programmed cell death in the floral nectary of *Digitalis purpurea*. *Annals of Botany*.

[78] Lam E (2004). Controlled cell death, plant survival and development. *Nature Reviews Molecular Cell Biology*.

[79] Liu Y, Schiff M, Czymmek K, Tallóczy Z, Levine B, Dinesh-Kumar SP (2005). Autophagy regulates programmed cell death during the plant innate immune response. *Cell*.

[80] Patel S, Dinesh-Kumar SP (2008). *Arabidopsis* ATG6 is required to limit the pathogen-associated cell death response. *Autophagy*.

[81] Su W, Ma H, Liu C, Wu J, Yang J (2006). Identification and characterization of two rice autophagy associated genes, OsAtg8 and OsAtg4. *Molecular Biology Reports*.

[82] Ashford TP, Porter KR (1962). Cytoplasmic components in hepatic cell lysosomes. *The Journal of Cell Biology*.

[83] Fengsrud M, Erichsen ES, Berg TO, Raiborg C, Seglen PO (2000). Ultrastructural characterization of the delimiting membranes of isolated autophagosomes and amphisomes by freeze-fracture electron microscopy. *European Journal of Cell Biology*.

[84] Klionsky DJ, Abeliovich H, Agostinis P (2008). Guidelines for the use and interpretation of assays for monitoring autophagy in higher eukaryotes. *Autophagy*.

[85] Eskelinen EL (2008). To be or not to be? Examples of incorrect identification of autophagic compartments in conventional transmission electron microscopy of mammalian cells. *Autophagy*.

[86] Birmingham CL, Canadien V, Gouin E (2007). Listeria monocytogenes evades killing by autophagy during colonization of host cells. *Autophagy*.

[87] Mayhew TM (2007). Quantitative immunoelectron microscopy: alternative ways of assessing subcellular patterns of gold labeling. *Methods in Molecular Biology*.

[88] He P, Peng Z, Luo Y (2009). High-throughput functional screening for autophagy-related genes and identification of TM9SF1 as an autophagosome-inducing gene. *Autophagy*.

[89] Phillips AR, Suttangkakul A, Vierstra RD (2008). The ATG12-conjugating enzyme ATG10 is essential for autophagic vesicle formation in *Arabidopsis thaliana*. *Genetics*.

[90] Ishida H, Yoshimoto K, Izumi M (2008). Mobilization of Rubisco and stroma-localized fluorescent proteins of chloroplasts to the vacuole by an ATG gene-dependent autophagic process. *Plant Physiology*.

[91] Shvets E, Fass E, Elazar Z (2008). Utilizing flow cytometry to monitor autophagy in living mammalian cells. *Autophagy*.

[92] Cummins I, Steel PG, Edwards R (2007). Identification of a carboxylesterase expressed in protoplasts using fluorescence-activated cell sorting. *Plant Biotechnology Journal*.

[93] Mäe M, Myrberg H, Jiang Y, Paves H, Valkna A, Langel U (2005). Internalisation of cell-penetrating peptides into tobacco protoplasts. *Biochimica et Biophysica Acta*.

[94] Yao N, Eisfelder BJ, Marvin J, Greenberg JT (2004). The mitochondrion—an organelle commonly involved in programmed cell death in *Arabidopsis thaliana*. *The Plant Journal*.

[95] Mizushima N, Yoshimori T (2007). How to interpret LC3 immunoblotting. *Autophagy*.

[96] Kuma A, Matsui M, Mizushima N (2007). LC3, an autophagosome marker, can be incorporated into protein aggregates independent of autophagy: caution in the interpretation of LC3 localization. *Autophagy*.

[97] Suzuki K, Kirisako T, Kamada Y, Mizushima N, Noda T, Ohsumi Y (2001). The pre-autophagosomal structure organized by concerted functions of APG genes is essential for autophagosome formation. *The EMBO Journal*.

[98] Shvets E, Elazar Z (2008). Autophagy-independent incorporation of GFP-LC3 into protein aggregates is dependent on its interaction with p62/SQSTM1. *Autophagy*.

[99] Ueno T, Sato W, Horie Y (2008). Loss of Pten, a tumor suppressor, causes the strong inhibition of autophagy without affecting LC3 lipidation. *Autophagy*.

[100] Giménez-Xavier P, Francisco R, Platini F, Pérez R, Ambrosio S (2008). LC3-I conversion to LC3-II does not necessarily result in complete autophagy. *International Journal of Molecular Medicine*.

[101] Yue Z, Jin S, Yang C, Levine AJ, Heintz N (2003). Beclin 1, an autophagy gene essential for early embryonic development, is a haploinsufficient tumor suppressor. *Proceedings of the National Academy of Sciences of the United States of America*.

[102] Pattingre S, Tassa A, Qu X (2005). Bcl-2 antiapoptotic proteins inhibit Beclin 1-dependent autophagy. *Cell*.

[103] Vieira OV, Botelho RJ, Rameh L (2001). Distinct roles of class I and class III phosphatidylinositol 3-kinases in phagosome formation and maturation. *The Journal of Cell Biology*.

[104] Kim DH, Eu YJ, Yoo CM (2001). Trafficking of phosphatidylinositol 3-phosphate from the trans-Golgi network to the lumen of the central vacuole in plant cells. *The Plant Cell*.

[105] Matsunaga K, Saitoh T, Tabata K (2009). Two Beclin 1-binding proteins, Atg14L and Rubicon, reciprocally regulate autophagy at different stages. *Nature Cell Biology*.

[106] Mizushima N, Kuma A, Kobayashi Y (2003). Mouse Apg16L, a novel WD-repeat protein, targets to the autophagic isolation membrane with the Apg12-Apg5 conjugate. *Journal of Cell Science*.

[107] Proikas-Cezanne T, Ruckerbauer S, Stierhof YD, Berg C, Nordheim A (2007). Human WIPI-1 puncta-formation: a novel assay to assess mammalian autophagy. *FEBS Letters*.

[108] Waddell S, Jenkins JR, Proikas-Cezanne T (2001). A “no-hybrids” screen for functional antagonizers of human p53 transactivator function: dominant negativity in fission yeast. *Oncogene*.

[109] Proikas-Cezanne T, Waddell S, Gaugel A, Frickey T, Lupas A, Nordheim A (2004). WIPI-1*α* (WIPI49), a member of the novel 7-bladed WIPI protein family, is aberrantly expressed in human cancer and is linked to starvation-induced autophagy. *Oncogene*.

[110] Ketteier R, Seed B (2008). Quantitation of autophagy by luciferase release assay. *Autophagy*.

[111] Tekinay T, Wu MY, Otto GP, Anderson OR, Kessin RH (2006). Function of the *Dictyostelium discoideum* Atg1 kinase during autophagy and development. *Eukaryotic Cell*.

[112] Lee SB, Kim S, Lee J (2007). ATG1, an autophagy regulator, inhibits cell growth by negatively regulating S6 kinase. *EMBO Reports*.

[113] Hara T, Takamura A, Kishi C (2008). FIP200, a ULK-interacting protein, is required for autophagosome formation in mammalian cells. *The Journal of Cell Biology*.

[114] Chan EYW, Longatti A, McKnight NC, Tooze SA (2009). Kinase-inactivated ULK proteins inhibit autophagy via their conserved C-terminal domains using an Atg13-independent mechanism. *Molecular and Cellular Biology*.

[115] Chung T, Suttangkakul A, Vierstra RD (2009). The ATG autophagic conjugation system in maize: ATG transcripts and abundance of the ATG8-lipid adduct are regulated by development and nutrient availability. *Plant Physiology*.

[116] Zatloukal K, Stumptner C, Fuchsbichler A (2002). p62 is a common component of cytoplasmic inclusions in protein aggregation diseases. *American Journal of Pathology*.

[117] Shvets E, Fass E, Scherz-Shouval R, Elazar Z (2008). The N-terminus and Phe52 residue of LC3 recruit p62/SQSTM1 into autophagosomes. *Journal of Cell Science*.

[118] Pankiv S, Clausen TH, Lamark T (2007). p62/SQSTM1 binds directly to Atg8/LC3 to facilitate degradation of ubiquitinated protein aggregates by autophagy. *The Journal of Biological Chemistry*.

[119] Pursiheimo JP, Rantanen K, Heikkinen PT, Johansen T, Jaakkola PM (2009). Hypoxia-activated autophagy accelerates degradation of SQSTM1/p62. *Oncogene*.

[120] Bjørkøy G, Lamark T, Brech A (2005). p62/SQSTM1 forms protein aggregates degraded by autophagy and has a protective effect on huntingtin-induced cell death. *The Journal of Cell Biology*.

[121] Harada M, Hanada S, Toivola DM, Ghori N, Omary MB (2008). Autophagy activation by rapamycin eliminates mouse Mallory-Denk bodies and blocks their proteasome inhibitor-mediated formation. *Hepatology*.

[122] Moriyasu Y, Hattori M, Jauh G-Y, Rogers JC (2003). Alpha tonoplast intrinsic protein is specifically associated with vacuole membrane involved in an autophagic process. *Plant and Cell Physiology*.

[123] Paglin S, Hollister T, Delohery T (2001). A novel response of cancer cells to radiation involves autophagy and formation of acidic vesicles. *Cancer Research*.

[124] Kanazawa T, Taneike I, Akaishi R (2004). Amino acids and insulin control autophagic proteolysis through different signaling pathways in relation to mTOR in isolated rat hepatocytes. *The Journal of Biological Chemistry*.

[125] Munafó DB, Colombo MI (2001). A novel assay to study autophagy: regulation of autophagosome vacuole size by amino acid deprivation. *Journal of Cell Science*.

[126] Takeuchi H, Kanzawa T, Kondo Y, Kondo S (2004). Inhibition of platelet-derived growth factor signalling induces autophagy in malignant glioma cells. *British Journal of Cancer*.

[127] Yu L, Wan F, Dutta S (2006). Autophagic programmed cell death by selective catalase degradation. *Proceedings of the National Academy of Sciences of the United States of America*.

[128] Biederbick A, Kern HF, Elsasser HP (1995). Monodansylcadaverine (MDC) is a specific in vivo marker for autophagic vacuoles. *European Journal of Cell Biology*.

[129] Bampton ET, Goemans CG, Niranjan D, Mizushima N, Tolkovsky AM (2005). The dynamics of autophagy visualized in live cells: from autophagosome formation to fusion with endo/lysosomes. *Autophagy*.

[130] Mizushima N (2004). Methods for monitoring autophagy. *The International Journal of Biochemistry & Cell Biology*.

[131] Seglen PO, Gordon PB, Poli A (1980). Amino acid inhibition of the autophagic/lysosomal pathway of protein degradation in isolated rat hepatocytes. *Biochimica et Biophysica Acta*.

[132] Seglen PO, Gordon PB (1982). 3-methyladenine: specific inhibitor of autophagic/lysosomal protein degradation in isolated rat hepatocytes. *Proceedings of the National Academy of Sciences of the United States of America*.

[133] Venerando R, Miotto G, Kadowaki M, Siliprandi N, Mortimore GE (1994). Multiphasic control of proteolysis by leucine and alanine in the isolated rat hepatocyte. *American Journal of Physiology*.

[134] Weckwerth W, Wenzel K, Fiehn O (2004). Process for the integrated extraction, identification and quantification of metabolites, proteins and RNA to reveal their co-regulation in biochemical networks. *Proteomics*.

[135] Nelson CJ, Huttlin EL, Hegeman AD, Harms AC, Sussman MR (2007). Implications of ^15^N-metabolic labeling for automated peptide identification in *Arabidopsis thaliana*. *Proteomics*.

[136] Engelsberger WR, Erban A, Kopka J, Schulze WX (2006). Metabolic labeling of plant cell cultures with K^15^NO_3_ as a tool for quantitative analysis of proteins and metabolites. *Plant Methods*.

[137] Gruhler A, Schulze WX, Matthiesen R, Mann M, Jensen ON (2005). Stable isotope labeling of *Arabidopsis thaliana* cells and quantitative proteomics by mass spectrometry. *Molecular & Cellular Proteomics*.

[138] Gordon PB, Tolleshaug H, Seglen PO (1985). Use of digitonin extraction to distinguish between autophagic-lysosomal sequestration and mitochondrial uptake of [^14^C]sucrose in hepatocytes. *Biochemical Journal*.

[139] Gordon PB, Høyvik H, Seglen PO (1985). Sequestration and hydrolysis of electroinjected [^14^C]lactose as a means of investigating autophagosome-lysosome fusion in isolated rat hepatocytes. *Progress in Clinical and Biological Research*.

[140] Barnett JA, Payne RW, Yarrow D (1983). *Yeasts: Characteristics and Identification*.

[141] Klionsky DJ (2007). Monitoring autophagy in yeast: the Pho8Delta60 assay. *Protein Targeting Protocols*.

[142] Ishida H, Yoshimoto K (2008). Chloroplasts are partially mobilized to the vacuole by autophagy. *Autophagy*.

[143] Furuya N, Kanazawa T, Fujimura S, Ueno T, Kominami E, Kadowaki M (2001). Leupeptin-induced appearance of partial fragment of betaine homocysteine methyltransferase during autophagic maturation in rat hepatocytes. *The Journal of Biochemistry*.

[144] Nimmerjahn F, Milosevic S, Behrends U (2003). Major histocompatibility complex class II-restricted presentation of a cytosolic antigen by autophagy. *European Journal of Immunology*.

[145] Taylor GS, Long HM, Haigh TA, Larsen M, Brooks J, Rickinson AB (2006). A role for intercellular antigen transfer in the recognition of EBV-transformed B cell Lines by EBV nuclear antigen-specific CD4^+^ T cells. *The Journal of Immunology*.

[146] Rodriguez-Enriquez S, He L, Lemasters JJ (2004). Role of mitochondrial permeability transition pores in mitochondrial autophagy. *The International Journal of Biochemistry & Cell Biology*.

[147] Kanki T, Klionsky DJ (2008). Mitophagy in yeast occurs through a selective mechanism. *The Journal of Biological Chemistry*.

[148] Xue L, Fletcher GC, Tolkovsky AM (2001). Mitochondria are selectively eliminated from eukaryotic cells after blockade of caspases during apoptosis. *Current Biology*.

[149] Journet EP, Bligny R, Douce R (1986). Biochemical changes during sucrose deprivation in higher plant cells. *The Journal of Biological Chemistry*.

[150] Klionsky DJ (2007). Autophagy: from phenomenology to molecular understanding in less than a decade. *Nature Reviews Molecular Cell Biology*.

[151] Suzuki NN, Yoshimoto K, Fujioka Y, Ohsumi Y, Inagaki F (2005). The crystal structure of plant ATG12 and its biological implication in autophagy. *Autophagy*.

[152] Fujiki Y, Yoshimoto K, Ohsumi Y (2007). An *Arabidopsis* homolog of yeast *ATG6/VPS30* is essential for pollen germination. *Plant Physiology*.

[153] Harrison-Lowe NJ, Olsen LJ (2008). Autophagy protein 6 (ATG6) is required for pollen germination in *Arabidopsis thaliana*. *Autophagy*.

[154] Xiong Y, Contento AL, Bassham DC (2005). AtATG18a is required for the formation of autophagosomes during nutrient stress and senescence in *Arabidopsis thaliana*. *The Plant Journal*.

